# Lysophosphatidic Acid May Be a Novel Biomarker for Early Acute Aortic Dissection

**DOI:** 10.3389/fsurg.2021.789992

**Published:** 2022-01-10

**Authors:** Xiaogao Pan, Yang Zhou, Guifang Yang, Zhibiao He, Hongliang Zhang, Zhenyu Peng, Wen Peng, Tuo Guo, Mengping Zeng, Ning Ding, Xiangping Chai

**Affiliations:** ^1^Department of Emergency Medicine, Second Xiangya Hospital, Central South University, Changsha, China; ^2^Emergency Medicine and Difficult Diseases Institute, Second Xiangya Hospital, Central South University, Changsha, China; ^3^Emergency Department, Changsha Central Hospital, University of South China, Changsha, China

**Keywords:** aortic dissection, biomarker, lysophosphatidic acid, diagnosis, chest pain

## Abstract

**Background:** Misdiagnosis and delayed diagnosis of acute aortic dissection (AAD) significantly increase mortality. Lysophosphatidic acid (LPA) is a biomarker related to coagulation cascade and cardiovascular-injury. The extent of LPA elevation in AAD and whether it can discriminate sudden-onset of acute chest pain are currently unclear.

**Methods:** We measured the plasma concentration of LPA in a cohort of 174 patients with suspected AAD chest pain and 30 healthy participants. Measures to discriminate AAD from other acute-onset thoracalgia were compared and calculated.

**Results:** LPA was significantly higher in AAD than in the AMI, PE, and the healthy (344.69 ± 59.99 vs. 286.79 ± 43.01 vs. 286.61 ± 43.32 vs. 96.08 ± 11.93, *P* < 0.01) within 48 h of symptom onset. LPA level peaked at 12 h after symptom onset, then gradually decreased from 12 to 48 h in AAD. LPA had an AUC of 0.85 (0.80–0.90), diagnosis threshold of 298.98 mg/dl, a sensitivity of 0.81, specificity of 0.77, and the negative predictive value of 0.85. The ROC curve of LPA is better than D-dimer (*P* = 0.041, Delong test). The decision curve showed that LPA had excellent standardized net benefits.

**Conclusion:** LPA showed superior overall diagnostic performance to D-dimer in early AAD diagnosis may be a potential biomarker, but additional studies are needed to determine the rapid and cost-effective diagnostic tests in the emergency department.

## Introduction

Aortic dissection is a life-threatening cardiovascular disease that causes ~10,000 deaths in the United States each year (21% before admission and 32% in-hospital) (1, 2). Recently, Sweden indicated that the incidence has increased about 7.2/100,000 (3). However, the early identification and diagnosis of high-risk chest pain as acute aortic dissection (AAD) is the major challenge. Treatment measures include coronary angiography and thrombolytic drugs, which may cause poor prognosis in 24.8% of AAD, who are misdiagnosed as acute myocardial infarction (AMI) or acute pulmonary embolism (PE) (4–6). Not only that, chest CT or MRI is time-money-consuming and limited by emergency room conditions compared to biomarkers for the diagnosis of AAD (7, 8).

Several researches have investigated AAD potential biomarkers for faster and more accurate clinical treatment, such as smooth muscle myosin (9), calcium binding protein (10), soluble elastin fragments (11), soluble ST2 (5), and D-dimer (12). While increased values for D-dimers raise suspicion for AAD (4), it is difficult to use it to desciminate AAD from PE in daily practice, and there are nine (2.43%) exhibited negative D-dimer results in 370 AAD patients according to our previous studies (13). Moreover, the younger and smaller thrombosis groups have low specificity. A valuable diagnostic marker should provide information for early identification or elimination to improve the clinical treatment of AAD (12).

Lysophosphatidic acid (LPA) is a small and simple glycerophospholipid (1-acyl-2-hydroxy-3-phosphoglycerol structure) (14), which is an early molecular marker of coagulation cascade activation and cardiovascular-injury (15, 16). Previous researches have shown that lysophosphatidylcholine (LPC) was also involved in aortic aneurysm formation and decreased substantially in AAD, which is hydrolyzed to produce LPA (17, 18). The LPA may be produced earlier than D-dimer, or have a better diagnostic performance than D-dimer and AAD risk scoring system (19, 20), but the relationship between LPA and AAD is unclear. The plasma LPA levels of patients with acute chest pain (AAD, AMI, PE) and healthy participants were measured and compared to evaluate the diagnostic performance in distinguishing AAD from other chest pains.

## Methods

### Research Sample

This is a single-center retrospective cohort including patients with suspected AAD within 48 h of onset and healthy participants, who came from the emergency department and medical examination center of the Second Xiangya Hospital of Central South University (Changsha, China) between May 2020 and January 2021. All suspected AAD patients were examined for medical imaging and D-dimer for the final diagnosis (19, 21). The exclusion criteria are shown in Supplementary Table 1.

About 3–5 ml of whole blood was taken from the brachial vein and placed in a sodium citrate anticoagulant tube immediately after hospital admission. The samples were centrifuged at 1000 r/min for 15 min to process into plasma, and stored at −80°C. All sample processing methods are similar.

The Ethics Committee of the Second Xiangya Hospital of Central South University approved this study. Informed consent was obtained from all patients. However, consent was obtained from a family member in a case of sudden death after admission or during autopsy.

### Outcome

All patients with AAD, characterized by symptoms onset-time within 48 h, had image information from aortic computed tomography to confirm the final diagnosis. AMI diagnosis criteria were: (1) chest pain lasting >20 min, (2) Serial ECG changes with new pathological Q waves or ST-segment and T-wave changes, and (3) a plasma creatine kinase-myocardial band elevation (more than twice the normal level or cardiac troponin I (cTnI) level > 0.1 ng/ml). A positive pulmonary artery computed tomography scan was for PE diagnosis.

### Measurement of LPA

The processed serum was measured by the human lysophosphatidic acid kit (Wuhan Huamei Bioengineering Co., Ltd.), and D-dimer was detected by the TOP700 automatic coagulation analyzer.

### Statistical Analysis

Continuous variables were expressed as mean ± standard deviation or median (IQR). ANOVA and Kruskal-Wallis test were used for parametric and non-parametric data in multiple groups, *T*-test and Mann-Whitney U-test were used as a *post-hoc* analysis. Categorical variables were expressed as frequencies and compared using Fisher's precision probability test or Chi-square analysis. Logistic regression analysis was also used. *P* = 0.05 was considered statistically significant. Pearson correlation and delong test (22) were used to compare the relationship and ROC curve between LPA and D-dimer. The decision curve for a theoretical distribution was given to describe expected net benefit (23, 24).

The R (https://www.r-project.org/), The R Foundation, and EmpowerStats (http://www.empowerstats.com), X&Y Solutions Inc, Boston, MA were used for all statistical analyses.

## Results

**Figure 1:**
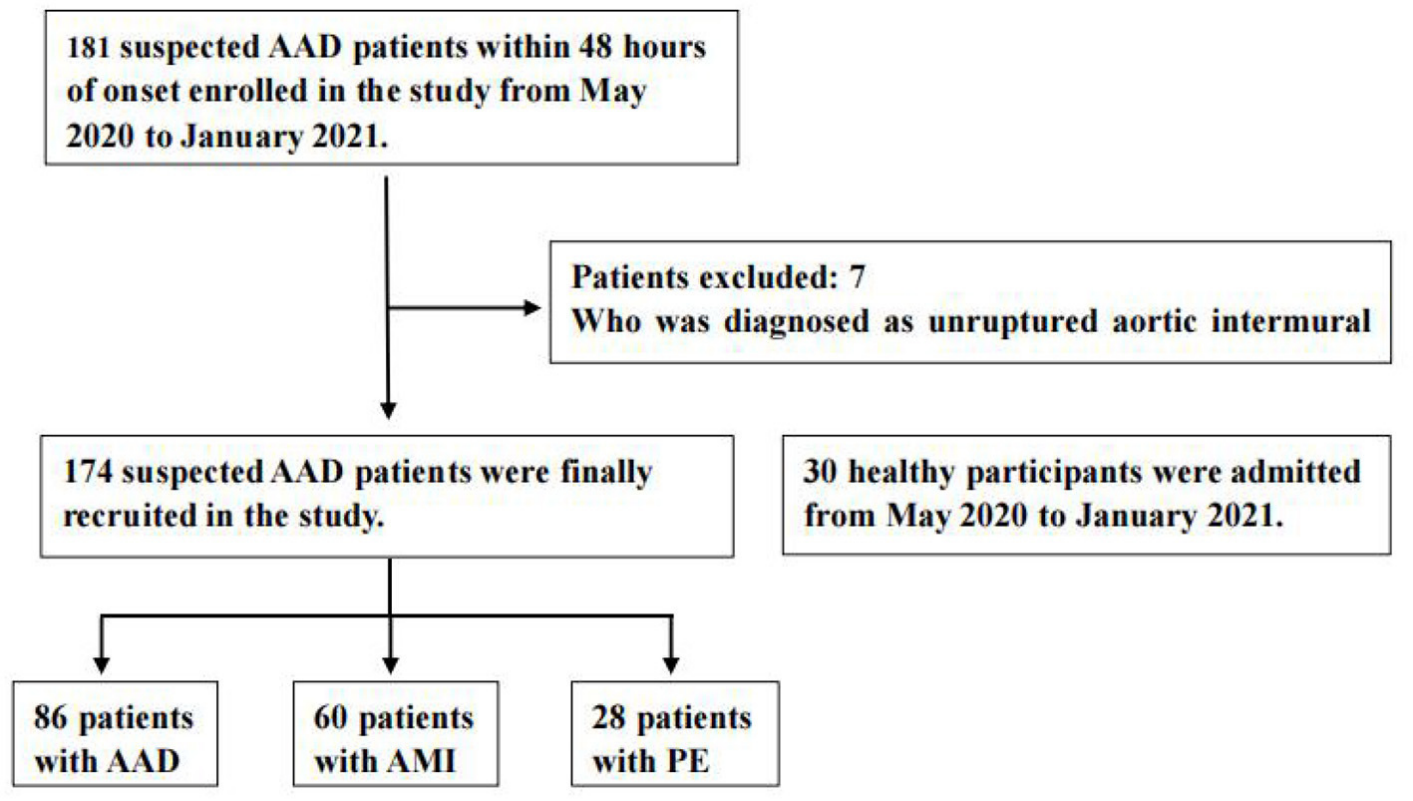
Flow diagram of exclusion and enrollment of study patients. Describes the exclusion and enrollment of study patients. AAD, acute aortic dissection; AMI, acute myocardial infarction; PE, pulmonary embolism.

**Figure 2:**
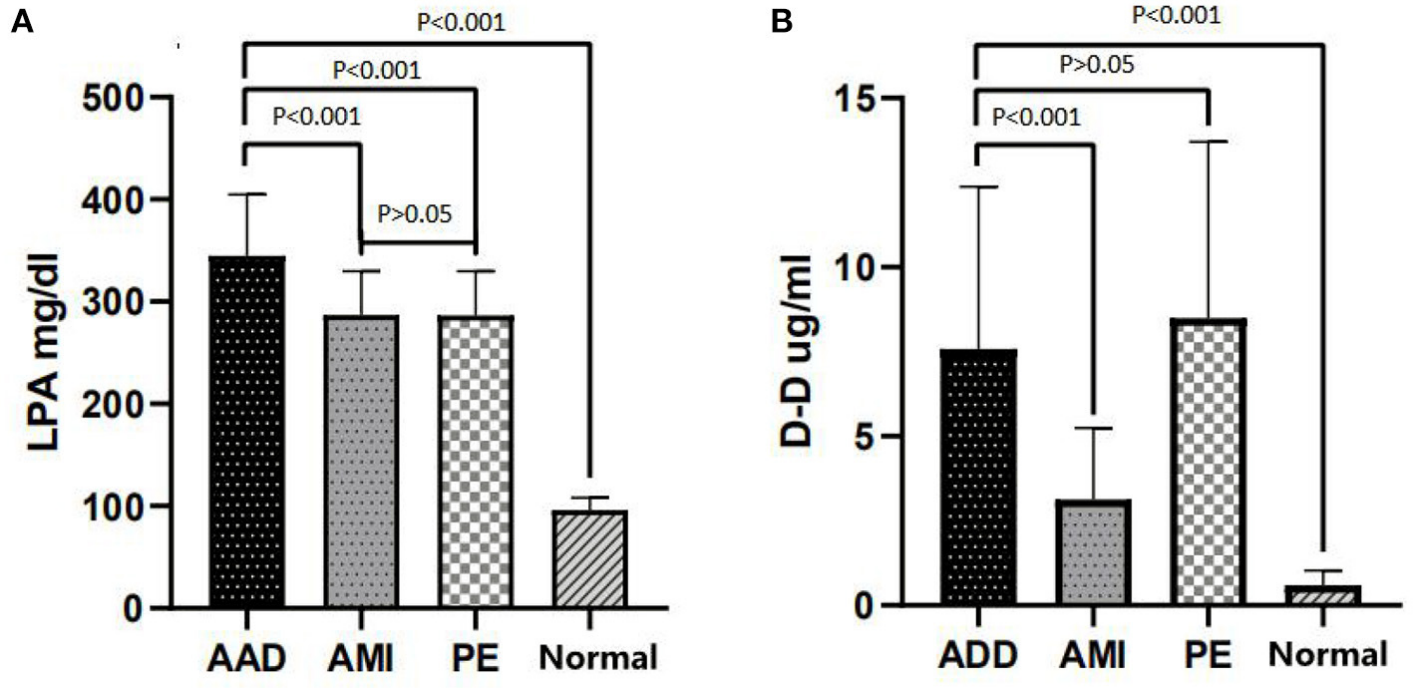
LPA and D-dimer levels in chest pain patients with AAD vs. other groups. **(A)** LPA distribution (Mean ± standard deviation) in AAD, AMI, PE, and Normal. **(B)** D-dimer distribution (Mean ± standard deviation) in AAD, AMI, PE, and Normal. LPA, lysophosphatidic acid; AAD, acute aortic dissection; AMI, acute myocardial infarction; PE, pulmonary embolism; Normal, healthy participants.

A total of 204 patients, including 86 AAD patients, 60 AMI patients, 28 PE patients, and 30 healthy participants (Normal), were selected (Figure 1). The patient characteristics after 48 h of onset are shown in Table 1 and Supplementary Table 2. AAD patients were mostly younger males with no significant difference in symptom onset time in all groups than non-AAD patients (Supplementary Table 3). The D-dimer and LPA levels were significantly higher in AAD patients than in non-AAD patients on the whole (Supplementary Table 2). LPA level (344.69 ± 59.99) was significantly different from AMI, PE, and Normal. However, AMI and PE were not statistically different (Figure 2A). D-dimer levels in AAD and PE were higher but no statistical difference between the two (Figure 2B). Logistic multiple regression analysis showed that the blood pressure differences, hypertension history, D-dimer, and LPA were independently associated with AAD (*P* < 0.001) (Table 2).

**Table 1:** Baseline characteristics of chest pain patients with AAD vs. other groups.

	**AAD**	**AMI**	**PE**	**Normal**	***P*-value**
No. of participates	86	60	28	30	
Gender, male	58 (67.44)^#^	32 (53.33)	12 (42.86)^†^	16 (53.33)	0.087
Age, year	53.60 ± 11.46^※^	57.30 ± 5.34^†^	55.36 ± 5.15	54.67 ± 4.33	0.077
Onset time to hospital, hours	11.45 ± 5.12	10.00 ± 4.43	11.32 ± 3.84	-	0.168
HR,/min	81.33 ± 18.22	79.57 ± 10.57	81.57 ± 10.82	78.37 ± 11.38	0.722
SBP, mmHg					
Left-S	141.21 ± 38.62	136.25 ± 15.99	136.32 ± 12.58	135.07 ± 15.03	0.608
Right-S	135.58 ± 36.93	135.40 ± 15.71	136.32 ± 13.21	135.50 ± 14.39	0.998
Difference-S	18.00 (7.00–31.00)^※^^#^^*^	3.00 (2.00–5.00)^†^	2.00 (2.00–4.00)^†^	3.00 (2.00–4.00)^†^	<0.001
DBP, mmHg					
Left-D	79.90 ± 22.31	83.42 ± 11.87	84.25 ± 9.60	79.83 ± 13.53	0.473
Right-D	76.94 ± 20.02^※^	83.83 ± 12.06^†^	84.96 ± 11.35	81.63 ± 13.25	0.030
Difference-D	10.00 (4.00–13.75)^※^^#^^*^	3.00 (2.00–4.25)^†^	3.00 (1.75–6.00)^†^	3.00 (2.00–5.00)^†^	<0.001
History of					
Hypertension, %	72 (83.72)^※^^#^^*^	25 (41.67)^†^^#^^*^	7 (25.00)^†^^※^	5 (16.67)^†^^※^^#^	<0.001
Diabetes, %	5 (5.81)^※^^#^	17 (28.33)^†^^#^^*^	5 (17.86)^†^	3 (10.00)^※^	0.002
Stroke, %	6 (6.98)	4 (6.67)	2 (7.14)	0 (0)	0.530
Chronic kidney disease, %	12 (13.95)	12 (20.00)^#^	3 (10.71)^*^	0 (0)	0.067
OSAS, %	26 (30.23)^※^^*^	0 (0)^†^^#^	7 (25.00)^※^^*^	0 (0)^†^^#^	<0.001
COPD, %	5 (5.81)	2 (3.33)	3 (10.71)	0 (0)	0.257
Marfan, %	2 (2.33)	0 (0)	0 (0)	0 (0)	0.428
CAD, %	10 (11.63)^※^^*^	26 (43.33)^†^^#^^*^	5 (17.86)^※^^*^	0 (0)^†^^※^^#^	<0.001
Valvular heart disease, %	2 (2.33)^※^^#^	8 (13.33)^†^^*^	3 (10.71)^†^^*^	0 (0)^※^^#^	0.017
Smoking, %	49 (56.98)^#^^*^	27 (45.00)^#^^*^	4 (14.29)^†^^※^	7 (23.33)^†^^※^	<0.001
Drinking, %	21 (24.42)^#^	11 (18.33)	3 (10.71)^†^^*^	6 (20.00)^#^	0.056
Medication history					
Aspirin, %	12 (13.95)*	11 (18.33)	5 (17.86)	0 (0)	0.100
Clopidogrel, %	7 (8.14)*	9 (15.00)	3 (10.71)	0 (0)	0.134
Statin, %	16 (18.60)*	10 (16.67)	3 (10.71)	0 (0)	0.075
Hormone, %	3 (3.49)*	2 (3.33)	1 (3.57)	0 (0)	0.784
D-dimer, ug/ml	7.58 ± 4.80^※^^*^	3.14 ± 2.11^†^^#^^*^	8.49 ± 5.22^※^^*^	0.59 ± 0.43^†^※^#^	<0.001
LPA, mg/dl	344.69 ± 59.99^※^^#^^*^	286.79 ± 43.01^†^^*^	286.61 ± 43.32^†^^*^	96.08 ± 11.93^†^^※^^#^	<0.001

*AAD, acute aortic dissection; AMI, acute myocardial infarction; PE, pulmonary embolism; SBP, systolic blood pressure; DBP, diastolic blood pressure; OSAS, obstructive sleep apnea syndrome; COPD, chronic obstructive pulmonary disease; CAD, coronary artery disease; LPA, lysophosphatidic acid. P < 0.05, statistically different*.

† *Significance vs. “AAD,”*

※ *Significance vs. “AMI,”*

# *Significance vs. “PE,”*

* *Significance vs. “Normal.”*

**Table 2:** Multivariate regression analysis for AAD diagnosis.

**Exposure**	**Univariate analysis**			**Multi-factor analysis**		
	**OR**	**95% CI**	***P***	**OR**	**95% CI**	***P***
Difference-S, mmHg	1.56	1.33, 1.84	<0.001	1.42	1.13, 1.78	0.003
Difference-D, mmHg	1.41	1.26, 1.57	<0.001	1.20	0.87, 1.67	0.272
Right-D, mmHg	0.97	0.96, 0.99	0.005	0.93	0.87, 1.00	0.047
Hypertension, %	11.26	5.64, 22.49	<0.001	9.67	1.93, 48.32	0.006
Diabetes, %	0.23	0.08, 0.63	0.004	0.04	0.00, 0.48	0.011
CAD, %	0.37	0.17, 0.80	0.012	0.15	0.01, 2.32	0.176
Valvular heart disease, %	0.23	0.05, 1.07	0.062	0.67	0.07, 6.97	0.740
Smoking, %	2.79	1.57, 4.96	0.001	0.41	0.07, 2.48	0.334
Drinking, %	2.40	1.14, 5.05	0.021	0.96	0.18, 5.10	0.962
D-dimer, ug/ml	1.03	1.02, 1.03	<0.001	1.02	1.01, 1.03	0.043
LPA, mg/dl	1.23	1.13, 1.30	<0.001	1.21	1.06, 1.43	0.007

*AAD, acute aortic dissection; Difference-S, difference of systolic blood pressure; Difference-D, difference of diastolic blood pressure; Right-D, the right diastolic blood pressure; CAD, coronary artery disease; LPA, lysophosphatidic acid. P < 0.05, Statistically different*.

Pearson analysis showed that LPA level was positively associated with D-dimer, *p* < 0.05) (coefficient of 0.17 in AAD, 0.15 in AMI, and 0.24 in PE). There was no significant correlation in Normal (Supplementary Figure 2).

### LPA Distribution

The box plots were used to analyze LPA levels in AAD patient plasma at different onset times (Supplementary Figure 3). Based on the different onsets of symptoms in AAD patients, LPA seems to peak at 12 h and gradually decreased after 12 h, easing after 24 h (*P* < 0.05). The number of chest pain patients at different symptoms onset-time was not statistically significant (*P* = 0.148) (Supplementary Table 3).

### Diagnostic Performance for Discriminating AAD

**Figure 3:**
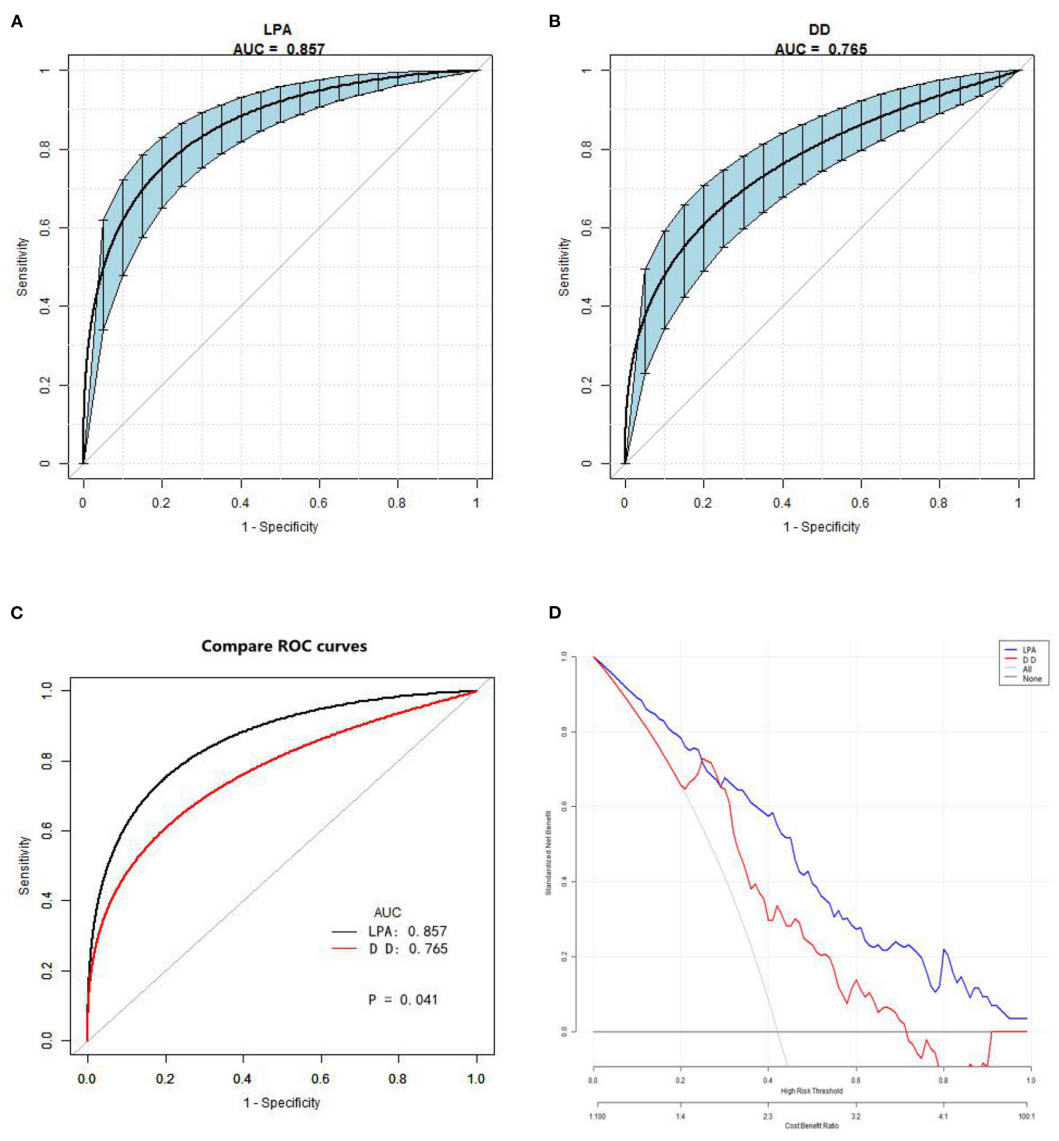
Receiver operating characteristic and decision curve. **(A)** The AUC value of LPA predicting AAD. **(B)** The AUC value of D-dimer predicting AAD. Blue shading shows the bootstrap estimated 95% CI with AUC. **(C)** Comparison of ROC curves between D-dimer and LPA. The ROC curve of LPA is better than D-dimer (Delong test). **(D)** Decision curve for a theoretical distribution. Solid line: prediction model, LPA = blue, DD = red. Thin gray line: assume all patients have AAD. Black bottom line: assume no patients have AAD. The vertical axis displays standardized net benefit. The two horizontal axes show the correspondence between risk threshold and cost:benefit ratio. The graph gives the expected net benefit per patient associated with LPA and DD, with LPA performing better. LPA, lysophosphatidic acid; AAD, acute aortic dissection; AUC, area under the curve.

The ROC analysis results indicated that D-dimer had an AUC of 0.76 (0.70–0.82), diagnosis threshold of 1.87 ug/ml, a sensitivity of 0.90, specificity of 0.55, and the negative predictive value of 0.91. LPA had an AUC of 0.85 (0.80–0.90), diagnosis threshold of 298.98 mg/dl, a sensitivity of 0.81, specificity of 0.77, and the negative predictive value of 0.85 (Table 3; Figures 3A,B). The ROC curve of LPA is better than D-dimer (*P* = 0.041, Delong test) (Figure 3C). The decision curve for a theoretical distribution showed that LPA had excellent standardized net benefits (Figure 3D).

**Table 3:** Diagnostic performance of AAD patients vs. others using LPA compared with D-Dimer.

	**AUC**	**95% CI**	**Threshold**	**Sensitivity**	**Specificity**	**PLR**	**NLR**	**PPV**	**NPV**
D-D, ug/ml	0.76	0.70–0.82	1.87	0.90	0.55	1.82	0.11	0.57	0.91
LPA, mg/dl	0.86	0.80–0.90	298.98	0.81	0.77	3.56	0.24	0.72	0.85
*P* for	0.041	-	-	-	-	-	-	-	-
compare									

*D-D, d-dimer; LPA, lysophosphatidic acid. P < 0.05, Statistically different (Delong test)*.

## Discussion

This study, which included 174 suspected AAD patients and 30 healthy participants, found that LPA may be used for the clinical diagnosis of AAD. LPA, mainly produced by activated platelets, may be an early biomarker of the initiation of thrombosis and coagulation. Researches have shown that coagulation cascade activates platelets to release a large amount of lysophospholipids and autotaxin with phospholipase D activity stored in alpha particles at the same time. The two substances undergo a biochemical reaction to produce LPA, which increases the plasma concentration (14, 25, 26). LPA binding to its receptors (LPA1-6) on platelet surface activates platelets and forms a positive feedback reaction, alters platelet in morphology, promotes aggregation and thrombus stability (27–29). Moreover, LPA activates LPA1 and LPA3 expressed in vascular endothelial cells (30), promotes immune response and aggravate endothelial cell damage via Gαi-RhoA-ROCK-NF-κB dependent pathways (31). LPA also increases the expression of intracellular matrix metalloproteinase-2, which will remodel the extracellular matrix and reduce the aggregation of endothelial cells to promote the migration and aggregation of inflammatory cells, thereby damaging endothelial cells to activate the coagulation cascade (32).

We found that the magnitude of elevated LPA can distinguish patients with AAD from patients with AMI, PE, and the healthy within 48 h after symptom onset. AAD is a disease with high mortality due to severe damage to the aortic structure. There are also some biomarkers showing clinical prognosis including CRP (33), NT-pro BNP (34) and cardiac troponin (35). When aortic dissection occurs, disruption to the aortic media immediately changes aorta hemodynamics, then intramural hematoma expand (especially when the intimal layer is also disrupted) as the blood flow inside media (5). The tissue coagulation factor III directly exposed to the blood in the arterial smooth muscle activates the exogenous coagulation pathway and promotes the coagulation cascade (13). Platelets, an indispensable role in the coagulation cascade, are continuously activated by the LPC-ATX-LPA pathway to produce more LPA before the aortic dissection is surgically repaired (14, 36). Although some studies speculate that the half-life of LPA is only 2–3 min under the action of LPPs, the continuous existence of disruption to the aortic media and positive feedback may be a reasonable explanation for the increase in plasma LPA in AAD patients (37, 38).

Our results suggested that the degree of the elevation of LPA levels is associated with the different magnitudes of vascular injury among AAD, AMI, and PE. Although LPA level in PE and AMI patients was higher than the healthy, there were still lower than AAD in our study. Substantial aortic hemodynamic changes significantly increased the circulating LPA level in the aorta (largest artery) compared with the small and medium blood vessels of pulmonary embolism and acute coronary syndrome. Studies have shown that LPA level is associated with the release location, releasing LPA directly and quickly to the aortic circulation may be another reason for the higher degree of AAD patients (39).

In addition, we speculated that LPA peaks at 12 h according to the symptoms of AAD patients, and our results also found that the threshold of LPA is 298.98 mg/dl, the specificity and AUC are 0.77 and 0.857, respectively, which are better than D-dimer (*P* < 0.05, Delong test). This means that as a marker of platelet activation, LPA ≥ 300 mg/dl indicates a high risk of AAD, and may be earlier than D-dimer. However, D-dimer has higher sensitivity and negative predictive value compared to LPA. As a fibrin degradation product in the circulation after thrombolytic fibrinolysis, D-dimer has been found to increase similarly in many diseases, including PE and AAD. In fact, D-dimer aids clinical diagnosis for PE only as a rule-out tool when the test result is negative (5).

To the best of our knowledge, this is the first study showing that LPA is associated with aortic dissection and could be a novel AAD biomarker. However, this study has some limitations. It is still unclear how LPA changes over time in AAD patients, a large-scale prospective multicenter study is needed to confirm the generalizability of the findings, the diagnostic validity, and the accuracy of this novel detection method since this was a single-center study. Second, the assay method used could provide an inconsistent absolute value for the LPA concentration, influencing the recommended cut-off level. Therefore, various detection methods should be accurately calibrated. Thirdly, this study did not include all undifferentiated chest pain patients, hence, patient selection could be biased. Finally, LPA <300 mg/dl is difficult to rule out AAD in daily practice, and other diseases (ovarian cancer) (40) can also increase LPA levels. Therefore, further confirmatory diagnosis based on medical images is essential to prevent misdiagnosis in clinical practice.

## Conclusion

LPA showed superior overall diagnostic performance to D-dimer in early AAD diagnosis may be a potential biomarker, but additional studies are needed to determine the rapid and cost-effective diagnostic tests in the emergency department.

## Data Availability Statement

The data analyzed in this study is subject to the following licenses/restrictions: We are deeply sorry for this, because there is still some research in progress for the time being. In order not to affect the following research, it is not convenient to disclose the data. Requests to access these datasets should be directed to 188212324@csu.edu.cn.

## Ethics Statement

The hospital institutional review board of the Second Xiangya Hospital approved the study. The data collection and analysis followed the Ethics Committee of the institution and the Declaration of Helsinki. The Ethics Committee of the institution reviewed the patient consents, and the data were used only for research purposes.

## Author Contributions

XP and XC: drafted, revised, and reviewed the article. YZ and GY: conducted statistical analysis. ZH, HZ, ZP, WP, and ND: reviewed and revised the manuscripts. TG and MZ: organized the database. All authors significantly contributed to the conception, study design, execution, data acquisition, analysis, interpretation, approved the final version, agreed on the journal, and are responsible for this study.

## Funding

The Key Research and Development Program of Hunan Province; Hunan Health and Family Planning Commission Project; Hunan Provincial Natural Science Foundation Project (Nos. 2019SK2022; 20200063; 2021JJ30923) supported this study.

## Conflict of Interest

The authors declare that the research was conducted in the absence of any commercial or financial relationships that could be construed as a potential conflict of interest.

## Publisher's Note

All claims expressed in this article are solely those of the authors and do not necessarily represent those of their affiliated organizations, or those of the publisher, the editors and the reviewers. Any product that may be evaluated in this article, or claim that may be made by its manufacturer, is not guaranteed or endorsed by the publisher.

## References

[1] Nienaber C Clough R Sakalihasan N Suzuki T Gibbs R Mussa F. Aortic dissection. Nat. Rev. Dis. Primers. (2016) 2:16071. 10.1038/nrdp.2016.53 27560366

[2] Mussa F Horton J Moridzadeh R Nicholson J Trimarchi S Eagle K. Acute aortic dissection and intramural hematoma: a systematic review. JAMA. (2016) 316:754–63. 10.1001/jama.2016.10026 27533160

[3] Smedberg C Steuer J Leander K Hultgren R. Sex differences and temporal trends in aortic dissection: a population-based study of incidence, treatment strategies, and outcome in Swedish patients during 15 years. Eur Heart J. (2020) 41:2430–8. 10.1093/eurheartj/ehaa446 32558879 PMC7340356

[4] Bossone Eduardo LaBounty Troy M Eagle Kim A. Acute aortic syndromes: diagnosis and management, an update. Eur Heart J. (2018) 39:739–49. 10.1093/eurheartj/ehx319 29106452

[5] Wang Y Tan X Gao H Yuan H Hu R Jia L. Magnitude of Soluble ST2 as a Novel Biomarker for Acute Aortic Dissection. Circulation. (2018) 137:259–69. 10.1161/CIRCULATIONAHA.117.030469 29146682

[6] Pourafkari L Tajlil A Ghaffari S Parvizi R Chavoshi M Kolahdouzan K et al. The frequency of initial misdiagnosis of acute aortic dissection in the emergency department and its impact on outcome. Internal Emerg Med. (2017) 12:1185–95. 10.1007/s11739-016-1530-7 27592236

[7] Pape L Awais M Woznicki E Suzuki T Trimarchi S Evangelista A et al. Presentation, diagnosis, and outcomes of acute aortic dissection: 17-year trends from the international registry of acute aortic dissection. J Am Coll Cardiol. (2015) 66:350–8. 10.1016/j.jacc.2015.05.029 26205591

[8] Tsai TT Trimarchi S Nienaber CA. Acute Aortic Dissection: Perspectives from the International Registry of Acute Aortic Dissection (IRAD). Eur J Vasc Endovasc Surg. (2009) 37:149–59. 10.1016/j.ejvs.2008.11.032 19097813

[9] Suzuki T Katoh H Watanabe M Kurabayashi M Hiramori K Hori S. Novel biochemical diagnostic method for aortic dissection. Results of a prospective study using an immunoassay of smooth muscle myosin heavy chain. Circulation. (1996) 93:1244–9. 10.1161/01.CIR.93.6.1244 8653847

[10] Suzuki T Distante A Zizza A Trimarchi S Villani M Salerno Uriarte J. Preliminary experience with the smooth muscle troponin-like protein, calponin, as a novel biomarker for diagnosing acute aortic dissection. Eur Heart J. (2008) 29:1439–45. 10.1093/eurheartj/ehn162 18436559

[11] Shinohara T. Soluble elastin fragments in serum are elevated in acute aortic dissection. Arterioscler Thromb Vasc Biol. (2003) 23:1839–44. 10.1161/01.ATV.0000085016.02363.80 12842847

[12] Suzuki T. Eagle KA. Biomarker-assisted diagnosis of acute aortic dissection. Circulation. (2018) 137:270–2. 10.1161/CIRCULATIONAHA.117.032048 29335286

[13] Yang G Peng W Zhou Y He H Chai X. Characteristics and prognosis of acute type A aortic dissection with negative D-dimer result. Am J Emerg Med. (2020) 38:1820–4. 10.1016/j.ajem.2020.05.055 32738476

[14] Zhao Y Hasse S Zhao C Bourgoin S. Targeting the autotaxin–Lysophosphatidic acid receptor axis in cardiovascular diseases. Biochem Pharmacol. (2019) 164:74–81. 10.1016/j.bcp.2019.03.035 30928673

[15] Moolenaar W. Lysophosphatidic acid, a multifunctional phospholipid messenger. J Biol Chem. (1995) 270:12949–52. 10.1074/jbc.270.22.12949 7768880

[16] Abdel-Latif A Heron P Morris A Smyth S. Lysophospholipids in coronary artery and chronic ischemic heart disease. Curr Opin Lipidol. (2015) 26:432–7. 10.1097/MOL.0000000000000226 26270808 PMC4617523

[17] Doppler C Arnhard K Dumfarth J Heinz K Messner B Stern C. Metabolomic profiling of ascending thoracic aortic aneurysms and dissections–implications for pathophysiology and biomarker discovery. PLoS ONE. (2017) 12:e0176727. 10.1371/journal.pone.0176727 28467501 PMC5415060

[18] Zhou X Wang R Zhang T Liu F Zhang W Wang G. Identification of lysophosphatidylcholines and sphingolipids as potential biomarkers for acute aortic dissection via serum metabolomics. Eur J Vasc Endovasc Surg. (2018) 57:434–41. 10.1016/j.ejvs.2018.07.004 30087010

[19] Erbel R Aboyans V Boileau C Bossone E Bartolomeo R Eggebrecht H. 2014 ESC Guidelines on the diagnosis and treatment of aortic diseases: document covering acute and chronic aortic diseases of the thoracic and abdominal aorta of the adult. The Task Force for the Diagnosis and Treatment of Aortic Diseases of the European Society of Cardiology (ESC). Eur Heart J. (2014) 35:2873–926. 10.1093/eurheartj/ehu281 25173340

[20] Nazerian P Mueller C Soeiro A Leidel B Salvadeo S Giachino F. Diagnostic accuracy of the aortic dissection detection risk score plus d-dimer for acute aortic syndromes: the ADvISED prospective multicenter study. Circulation. (2018) 137:250–8. 10.1161/CIRCULATIONAHA.117.029457 29030346

[21] Hiratzka L Bakris G Beckman J Bersin R Carr V Casey D. 2010 ACCF/AHA/AATS/ACR/ASA/SCA/SCAI/SIR/STS/SVM guidelines for the diagnosis and management of patients with Thoracic Aortic Disease: a report of the American College of Cardiology Foundation/American Heart Association Task Force on Practice Guidelines, American Association for Thoracic Surgery, American College of Radiology, American Stroke Association, Society of Cardiovascular Anesthesiologists, Society for Cardiovascular Angiography and Interventions, Society of Interventional Radiology, Society of Thoracic Surgeons, and Society for Vascular Medicine. Circulation. (2010) 121: e266–369. 10.1161/CIR.0b013e3181d4739e 20233780

[22] DeLong E DeLong D Clarke-Pearson D. Comparing the areas under two or more correlated receiver operating characteristic curves: a nonparametric approach. Biometrics. (1988) 44:837–45. 10.2307/2531595 3203132

[23] Vickers A Elkin E. Decision curve analysis: a novel method for evaluating prediction models. Med Decis Making. (2006) 26:565–74. 10.1177/0272989X06295361 17099194 PMC2577036

[24] Kerr K Brown M Zhu K Janes H. Assessing the clinical impact of risk prediction models with decision curves: guidance for correct interpretation and appropriate use. J Clin Oncol. (2016) 34:2534–40. 10.1200/JCO.2015.65.5654 27247223 PMC4962736

[25] Zhou Y Little P Ta H Xu S Kamato D. Lysophosphatidic acid and its receptors: pharmacology and therapeutic potential in atherosclerosis and vascular disease. Pharmacol Ther. (2019) 204:107404. 10.1016/j.pharmthera.2019.107404 31472182

[26] Leblanc R Lee S David M Bordet J Norman D Patil R. Interaction of platelet-derived autotaxin with tumor integrin αVβ3 controls metastasis of breast cancer cells to bone. Blood. (2014) 124:3141–50. 10.1182/blood-2014-04-568683 25277122 PMC4231421

[27] Retzer M Essler M. Lysophosphatidic acid-induced platelet shape change proceeds via Rho/Rho kinase-mediated myosin light-chain and moesin phosphorylation. Cell Signal. (2000) 12:645–8. 10.1016/S0898-6568(00)00108-X 11080616

[28] Siess W Zangl K Essler M Bauer M Brandl R Corrinth C. Lysophosphatidic acid mediates the rapid activation of platelets and endothelial cells by mildly oxidized low density lipoprotein and accumulates in human atherosclerotic lesions. Proc Natl Acad Sci U S A. (1999) 96:6931–6. 10.1073/pnas.96.12.6931 10359816 PMC22019

[29] Haserück N Erl W Pandey D Tigyi G Ohlmann P Ravanat C et al. The plaque lipid lysophosphatidic acid stimulates platelet activation and platelet-monocyte aggregate formation in whole blood: involvement of P2Y1 and P2Y12 receptors. Blood. (2004) 103:2585–92. 10.1182/blood-2003-04-1127 14645014

[30] Shimada H Rajagopalan L. Rho kinase-2 activation in human endothelial cells drives lysophosphatidic acid-mediated expression of cell adhesion molecules via NF-kappaB p65. J Biol Chem. (2010) 285:12536–42. 10.1074/jbc.M109.099630 20164172 PMC3282996

[31] Lin C Chen C Chen J Lee H. Lysophospholipids increase IL-8 and MCP-1 expressions in human umbilical cord vein endothelial cells through an IL-1-dependent mechanism. J Cell Biochem. (2006) 99:1216–32. 10.1002/jcb.20963 16795034

[32] Neidlinger N Larkin S Bhagat A Victorino G Kuypers F. Hydrolysis of phosphatidylserine-exposing red blood cells by secretory phospholipase A2 generates lysophosphatidic acid and results in vascular dysfunction. J Biol Chem. (2006) 281:775–81. 10.1074/jbc.M505790200 16278219

[33] Vrsalović M. Vrsalović Presečki A. Admission C-reactive protein and outcomes in acute aortic dissection: a systematic review. Croat Med J. (2019) 60:309–15. 10.3325/cmj.2019.60.309 31483116 PMC6734568

[34] Vrsalovic M Vrsalovic Presecki A Aboyans V. N-terminal pro-brain natriuretic peptide and short-term mortality in acute aortic dissection: a meta-analysis. Clin Cardiol. (2020) 43:1255–9. 10.1002/clc.23436 32735030 PMC7661642

[35] Vrsalovic M. Prognostic effect of cardiac troponin elevation in acute aortic dissection: a meta-analysis. Int J Cardiol. (2016) 214:277–8. 10.1016/j.ijcard.2016.03.230 27082771

[36] Smyth S Kraemer M Yang L Van Hoose P Morris A. Roles for lysophosphatidic acid signaling in vascular development and disease. Biochim Biophys Acta Mol Cell Biol Lipids. (2020) 1865:158734. 10.1016/j.bbalip.2020.158734 32376340

[37] Tomsig J Snyder A Berdyshev E Skobeleva A Mataya C Natarajan V. Lipid phosphate phosphohydrolase type 1 (LPP1) degrades extracellular lysophosphatidic acid *in vivo*. Biochem J. (2009) 419:611–8. 10.1042/BJ20081888 19215222 PMC2677185

[38] Salous A Panchatcharam M Sunkara M Mueller P Dong A Wang Y. Mechanism of rapid elimination of lysophosphatidic acid and related lipids from the circulation of mice. J Lipid Res. (2013) 54:2775–84. 10.1194/jlr.M039685 23948545 PMC3770090

[39] Dohi T Miyauchi K Ohkawa R Nakamura K Kurano M Kishimoto T. Increased lysophosphatidic acid levels in culprit coronary arteries of patients with acute coronary syndrome. Atherosclerosis. (2013) 229:192–7. 10.1016/j.atherosclerosis.2013.03.038 23664202

[40] Xu Y Shen Z Wiper D Wu M Morton R Elson P. Lysophosphatidic acid as a potential biomarker for ovarian and other gynecologic cancers. JAMA. (1998) 280:719–23. 10.1001/jama.280.8.719 9728644

