# Supplementary material for: Lysophosphatidic Acid May Be a Novel Biomarker for Early Acute Aortic Dissection
Source: Front Surg. 2022 Jan 10;8:789992. doi: 10.3389/fsurg.2021.789992 (PMC8784386; doi:10.3389/fsurg.2021.789992)
Supplement: Supplementary file 1 [file Data_Sheet_1.docx]

**Supplementary materials**

**Supplementary Tables**

**Table S1.** The diagnosis, inclusion, and exclusion criteria.

| **Diagnosis criteria:**  **1.** Acute aortic dissection (AAD) diagnosis was conducted via aortic CT. (The 2014 European Society of Cardiology Guidelines for the Diagnosis and Treatment of Arterial Diseases).  2. Acute myocardial infarction (AMI) diagnosis was conducted through coronary angiography or cardiology treatment (AAD was excluded) (The 2018 "Guidelines for the Diagnosis and Treatment of Acute ST-segment Elevation Myocardial Infarction").  3. Pulmonary embolism (PE) diagnosis were performed through pulmonary artery CT (The 2019 APE Diagnosis and Treatment Guidelines)  4. Normal patients were excluded from chest pain and other diseases associated with LPA, such as AAD, AMI, PE, tumors, and so on. |
| --- |
| **Inclusion criteria:** Undiagnosed chest pain patients, symptoms within 48 hours and over 18 years old. |
| **Exclusion criteria:** Patients diagnosed before admission. Patients with special chest pains: tumors, trauma. Pregnant women. Chronic inflammatory disease history: systemic lupus erythematous, rheumatoid arthritis. Patients with other bleeding disorders, fractures, surgery. Patients with severe infectious diseases and coagulation disorders. Incomplete research information includes patients who have not been tested for LPA or D-dimer, or whose medical record information is incomplete. |

**Table S2.** AAD vs Non-AAD.

|  | **All** | **AAD** | **Non-AAD** | **P-value** |
| --- | --- | --- | --- | --- |
| No. of participates | 204 | 86 | 118 | - |
| Gender, male | 118 (57.84%) | 58 (67.44%) | 60 (50.85%) | 0.018 |
| Age, year | 55.09 ± 8.48 | 53.60 ± 11.46 | 56.17 ± 5.15 | 0.033 |
| Onset time to hospital, hours | 10.39 ± 5.58 | 10.73 ± 6.54 | 10.06 ± 4.45 | 0.426 |
| HR rate | 80.41 ± 14.39 | 81.33 ± 18.22 | 79.74 ± 10.81 | 0.438 |
| **SBP, mmHg** |  |  |  |  |
| Left-S | 138.18 ± 27.55 | 141.21 ± 38.62 | 135.97 ± 14.89 | 0.028 |
| Right-S | 135.62 ± 26.38 | 135.58 ± 36.93 | 135.64 ± 14.70 | 0.871 |
| Difference-S | 11.94(2.00-12.00) | 23.86(7.00-31.00) | 3.25 (2.00-4.00) | <0.001 |
| **DBP, mmHg** |  |  |  |  |
| Left-D | 81.52 ± 17.07 | 79.90 ± 22.31 | 82.70 ± 11.86 | 0.247 |
| Right-D | 80.76 ± 16.24 | 76.94 ± 20.02 | 83.54 ± 12.17 | 0.004 |
| Difference-D | 6.92 (2.00-8.25) | 11.74(4.00-13.75) | 3.40(2.00-5.00) | <0.001 |
| **History of** |  |  |  |  |
| Hypertension, % | 109 (53.43%) | 72 (83.72%) | 37 (31.36%) | <0.001 |
| Diabetes, % | 30 (14.71%) | 5 (5.81%) | 25 (21.19%) | 0.002 |
| Stroke, % | 12 (5.88%) | 6 (6.98%) | 6 (5.08%) | 0.571 |
| Chronic kidney disease, % | 27 (13.24%) | 12 (13.95%) | 15 (12.71%) | 0.796 |
| OSAS, % | 33 (16.18%) | 26 (30.23%) | 7 (5.93%) | <0.001 |
| COPD, % | 10 (4.90%) | 5 (5.81%) | 5 (4.24%) | 0.607 |
| Marfan, % | 2 (0.98%) | 2 (2.33%) | 0 (0) | 0.177 |
| CAD, % | 41 (20.10%) | 10 (11.63%) | 31 (26.27%) | 0.010 |
| Valvular heart disease, % | 13 (6.37%) | 2 (2.33%) | 11 (9.32%) | 0.043 |
| Smoking, % | 87 (42.65%) | 49 (56.98%) | 38 (32.20%) | <0.001 |
| Drinking, % | 35 (17.16%) | 21 (24.42%) | 14 (11.86%) | 0.019 |
| **Medication history** |  |  |  |  |
| Aspirin, % | 28 (13.73%) | 12 (13.95%) | 16 (13.56%) | 0.936 |
| Clopidogrel, % | 19 (9.31%) | 7 (8.14%) | 12 (10.17%) | 0.622 |
| Statin, % | 29 (14.22%) | 16 (18.60%) | 13 (11.02%) | 0.125 |
| Hormone, % | 6 (2.94%) | 3 (3.49%) | 3 (2.54%) | 0.693 |
| D-dimer, ug/ml | 5.37 (1.56-8.20) | 7.58 (3.46-10.83) | 3.76 (0.89-5.23) | <0.001 |
| LPA, mg/dl | 283.13 ± 95.37 | 344.69 ± 59.99 | 238.26 ± 91.39 | <0.001 |

**Abbreviations:** AAD, acute aortic dissection; AMI, acute myocardial infarction; PE, pulmonary embolism; SBP, systolic blood pressure; DBP, diastolic blood pressure; OSAS, obstructive sleep apnea syndrome; COPD, chronic obstructive pulmonary disease; CAD, coronary artery disease; LPA, lysophosphatidic acid. P<0.05, Statistically different.

**Table S3.** Onset time of various chest pains.

|  | **<4**  **Hours** | **4-8**  **Hours** | **8-12**  **Hours** | **12-16 Hours** | **16-20 Hours** | **20-24 Hours** | **24-48 Hours** | **P-value** |
| --- | --- | --- | --- | --- | --- | --- | --- | --- |
| AAD | 5 (5.81%) | 14(16.28%) | 31(36.04%) | 20(23.26%) | 8 (9.30%) | 6 (6.98%) | 2 (2.33%) | 0.148 |
| AMI | 5 (8.33%) | 13(21.67%) | 19(31.66%) | 13(21.67%) | 6(10.00%) | 4 (6.67%) | 0 (0.00%) |  |
| PE | 1 (3.57%) | 6(21.43%) | 5 (17.86%) | 9(32.14%) | 2 (7.14%) | 1 (3.57%) | 4(14.29%) |  |

**Abbreviations:** AAD, acute aortic dissection; AMI, acute myocardial infarction; PE, pulmonary embolism.

**Supplementary Figures**

**
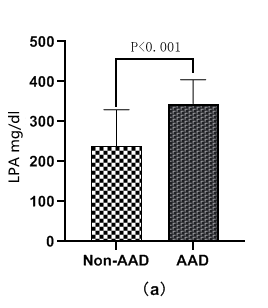

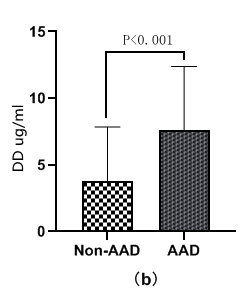
**

**Figure S1.** LPA and D-dimer levels in AAD and non-AAD. (a) LPA distribution (Mean±standard deviation) in AAD and non-AAD. (b) D-dimer distribution (Mean ±standard deviation) in AAD and non-AAD. LPA, Lysophosphatidic acid. DD, D-dimer.

**
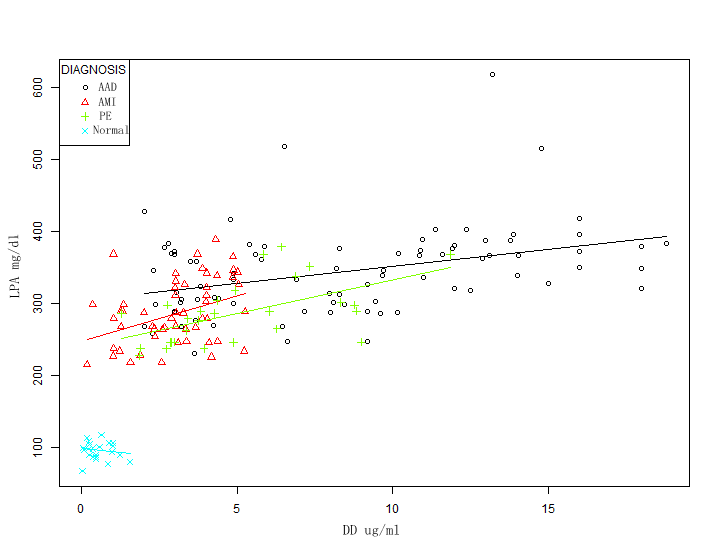
**

**Figure S2.** Pearson’s correlation coefficients based on the individual log-LPA and log-D-dimer measurements. LPA level was positively associated with D-dimer levels (P<0.05) (coefficient of 0.17 in AAD, 0.15 in AMI, and 0.24 in PE). There was no significant correlation in Normal.


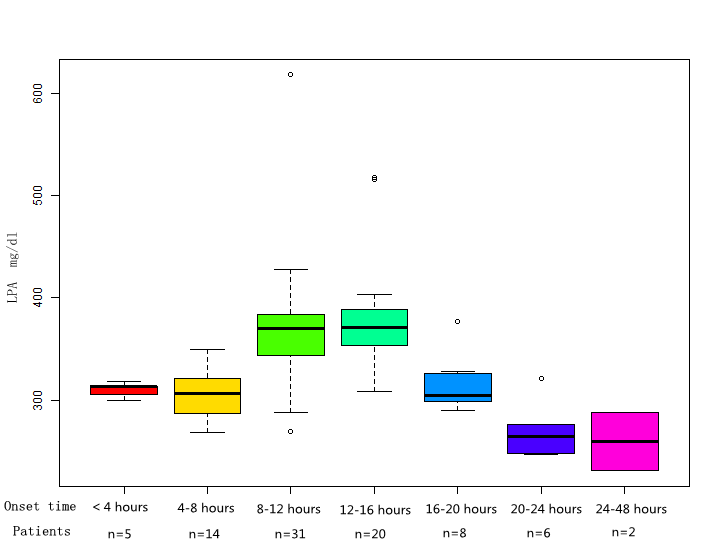


**Figure S3.** Boxplots of serum LPA analysis based on symptoms onset time in AAD patients (Median,IQR). LPA, lysophosphatidic acid.
